# Supplementary material for: An intra-oral flavor detection task in freely moving mice
Source: iScience. 2024 Jan 16;27(2):108924. doi: 10.1016/j.isci.2024.108924 (PMC10847684; doi:10.1016/j.isci.2024.108924)
Supplement: Document S1. Figures S1–S4 [file mmc1.pdf]

iScience, Volume *27*

Supplemental information

An intra-oral flavor detection task

in freely moving mice

Kazuki Shiotani, Yuta Tanisumi, Yuma Osako, Koshi Murata, Junya Hirokawa, Yoshio Sakurai, and Hiroyuki Manabe

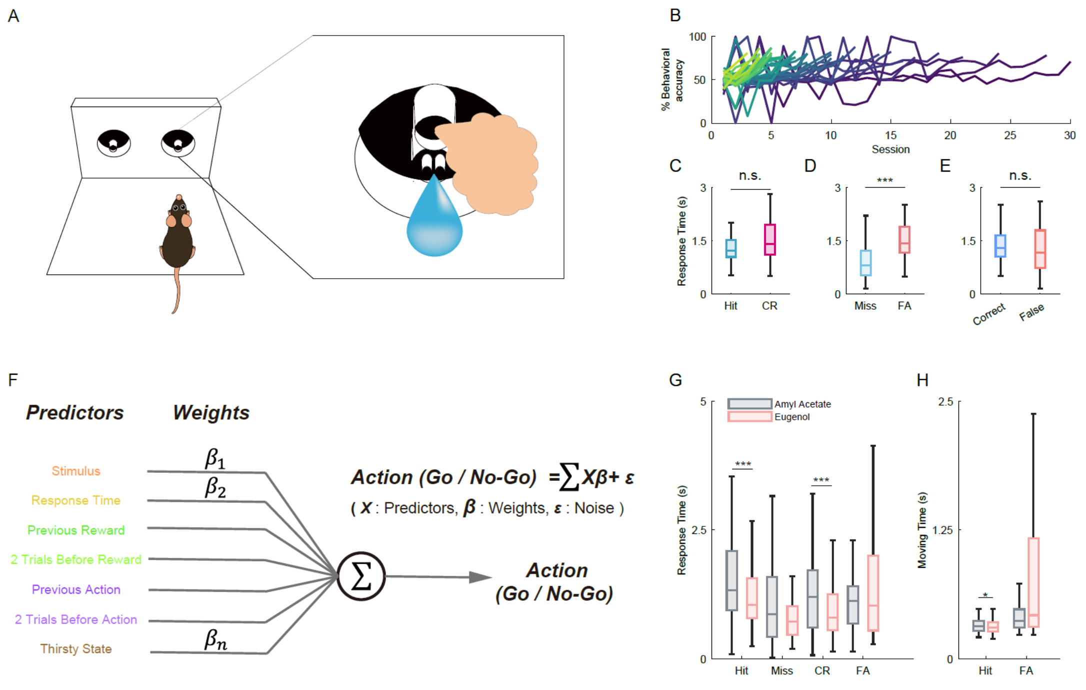

Supplementary Figure S1. Details of the flavor identification task and a schematic of the encoding model fitted to the behavioral variables, related to Figure 2.

(A) Expanded view of the stimulus port in the flavor detection task. The pipe of the external odor stimulus is attached to the top of that of the flavor stimulus.

(B) Performance of all mice in the flavor detection task. Each individual line represents one mouse.

(C) The boxplots of response times for Hit and correct rejection (CR) in the last training session (n.s., not significant; ***$p < 0.01$, Wilcoxon rank sum test).

(D) Same as in (C) but in Miss and false alarm (FA).

(E) Same as in (C) but in correct (Hit and CR) and false (Miss and FA).

(F) Schematic of the encoding model fitted to behavioral variables (see STAR Methods).

(G) The boxplot of response time for individual actions of the amyl acetate group (black) and the eugenol group (pink) sessions (***$p < 0.001$, Wilcoxon rank sum test).

(H) The boxplot of moving time for Hit and FA of the amyl acetate group (black) and the eugenol group (pink) (*$p < 0.05$, Wilcoxon rank sum test).

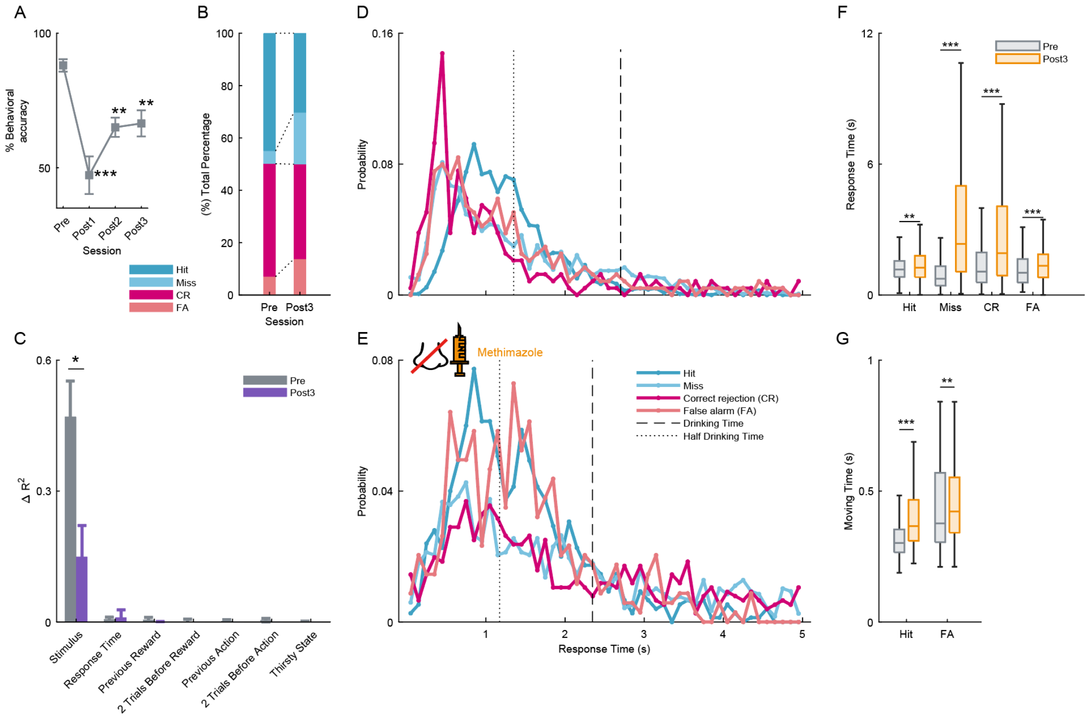

Supplementary Figure S2. Changes in the response time and moving time by intraperitoneal administration of methimazole, related to Figure 3.

(A) The time course of average behavioral accuracy after methimazole administration in the control group (gray line) (**p<0.01; ***p<0.001, Tukey's test). Error bars show SEM.

(B) Total percentage bar showing the proportion of the behavioral action in the pre and post3 sessions of the control group (blue, Hit; light blue, Miss; red, correct rejection [CR]; light red, false alarm [FA]).

(C) Effect of task parameters on behavioral variability with methimazole administration in the saline group using a GLM fitting for go or no-go actions (*p < 0.05, two-sample t-test, see STAR Methods).

(D) Histogram of the response time in the pre session.

(E) Same as in (D) but in post3 session.

(F) The average response time of Hit, Miss, CR and FA in the pre and post3 sessions of the methimazole group (**p < 0.01； ***p < 0.001, Wilcoxon rank sum test).

(G) The average moving time of Hit and FA in the pre and post3 sessions (**p < 0.01; ***p < 0.001, Wilcoxon rank sum test).

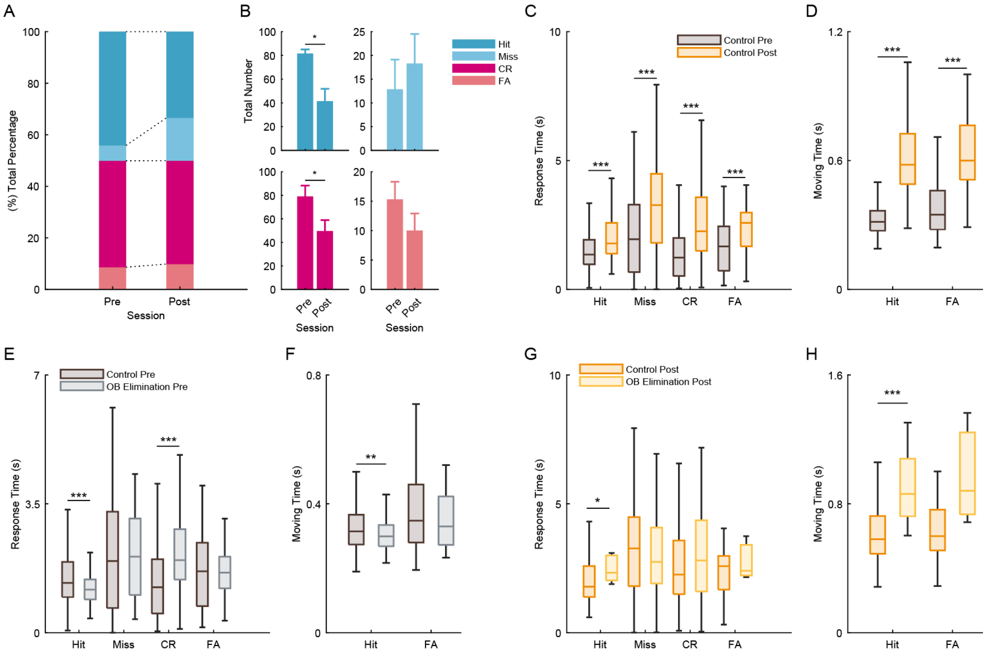

Supplementary Figure S3. Changes in behavioral responses, response time and moving time in the olfactory bulb removal and control groups, related to Figure 5.

(A) Total percentage bar showing the proportion of the behavioral action in the pre and post sessions of the control group (blue, Hit; light blue, Miss; red, correct rejection [CR]; light red, false alarm [FA]).

(B) The average of the total number bar showing the number of trials in which behavioral actions were taken of the control group. This number is the actual number used to calculate each condition in the total percentage bar (Figure S3A) (*p<0.05, paired t-test). Error bars show SEM.

(C) The boxplot of response time for individual actions in the pre (black) and post (orange) sessions of control group (***p < 0.001, Wilcoxon rank sum test).

(D) The boxplot of moving time for Hit and FA in the pre (black) and post (orange) sessions of control group (***p < 0.001, Wilcoxon rank sum test).

(E) Same as in (C) but in the pre sessions of control group (black) and olfactory bulb elimination group (gray) (***p < 0.001, Wilcoxon rank sum test).

(F) Same as in (D) but in the pre sessions of o control group (black) and olfactory bulb elimination group (gray) (**p < 0.01, Wilcoxon rank sum test).

(G) Same as in (C) but in the post sessions of control group (orange) and olfactory bulb elimination group (yellow) (*p < 0.05, Wilcoxon rank sum test).

(H) Same as in (D) but in the post sessions of control group (orange) and olfactory bulb elimination group (yellow (***p < 0.05, Wilcoxon rank sum test).

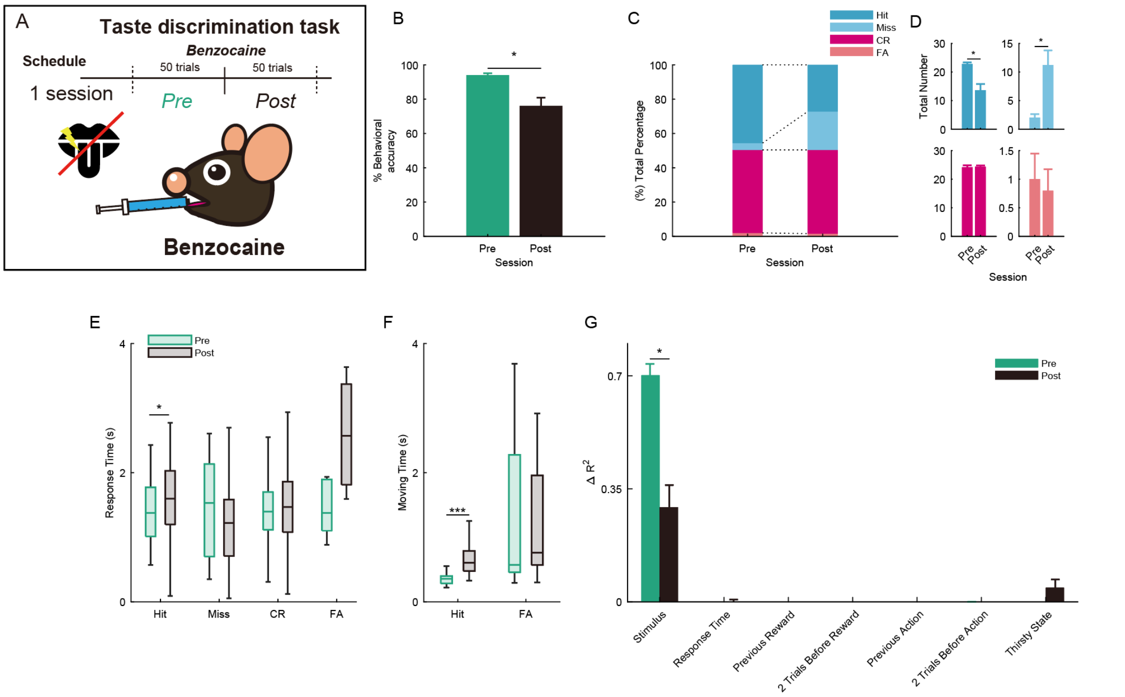

Supplementary Figure S4. Effects of benzocaine on the taste discrimination task, related to Figure 6.

(A) Schematic of the schedule of the benzocaine group (n=5 mice) in the taste discrimination task. The pre session (50 trials) represents the session before the benzocaine procedure. The post session (50 trials) represents the session immediately after the pre session, when benzocaine was administered to all the mice.

(B) The average behavioral accuracy in the pre (green) and post (black) sessions of the benzocaine procedure. The behavioral accuracy in the pre session was not significant compared to the post session (*$p < 0.05$, paired t-test). Error bars show SEM.

(C) Percentage bar showing the percentage of the behavioral action in the pre and post sessions (blue, Hit; light blue, Miss; red, correct rejection [CR]; light red, false alarm [FA]).

(D) The average of the total number bar showing the actual number of trials in which behavioral actions were taken and corresponding to the total percentage bar (Figure S4C) (*$p < 0.05$, paired t-test). Error bars show SEM.

(E) The boxplot of response time for individual actions in the pre (green) and post (black) sessions (*$p < 0.05$, Wilcoxon rank sum test).

(F) The boxplot of moving time for Hit and FA in the pre (green) and post (black) sessions (***$p < 0.001$, Wilcoxon rank sum test).

(G) Effect of task parameters on behavioral variability with benzocaine administration using a GLM fitting for go or no-go actions. Each average ΔR^2 (green, pre session; black, post session) is shown (*$p < 0.05$, paired t-test). Error bars show SEM.
